# Identification of Soy‐Derived Peptides With Micelle Disruption Activity of Secondary Bile Acids

**DOI:** 10.1002/fsn3.70319

**Published:** 2025-05-26

**Authors:** Shota Shimizu, Keita Hirano, Tsutomu Saito, Hirokazu Akiyama, Kazunori Shimizu, Hiroyuki Honda

**Affiliations:** ^1^ Department of Biomolecular Engineering, Graduate School of Engineering Nagoya University Nagoya Japan; ^2^ Research Institute for Creating the Future Fuji Oil Holdings Inc. Ibaraki Japan

## Abstract

Bile acids, which originate in the liver as primary bile acids, facilitate the absorption of lipophilic components into the intestines and exist as micellar structures. Intestinal bacteria convert primary bile acids to secondary bile acids, such as deoxycholic acid (DCA), which is associated with colorectal cancer. Therefore, their elimination from the body is preferred. Soy protein hydrolysates are known for their bile acid‐binding properties, which prompted our investigation of DCA‐specific binding and micelle‐disrupting peptides. We designed a peptide library from eight soy protein sequences and selected 42 candidate peptides based on their DCA‐binding activity. These peptides were synthesized, and their ability to disrupt bile acid micelles was assessed using a 96‐well high‐throughput assay system. Of the 42 peptides evaluated, 41 exhibited significant DCA micelle degradation, and 21 showed specific activity only for DCA micelles. Further research involved preparing soy protein pepsin hydrolysates and identifying 1,354 free peptides. Among these, 10 peptides containing sequences from the initial peptide library were identified. All peptides exhibited DCA micelle‐disrupting activity. These peptides are believed to facilitate the excretion of secondary bile acids. Notably, FGSLRKNAM and SLRKNAM selectively disrupted DCA micelles without affecting the micelles of other bile acids. As these two peptides were also identified in the undigested high molecular weight fraction, they are considered key peptides with high potential for efficiently promoting the excretion of DCA.

## Graphical abstract

DCA‐binding peptides were identified in a peptide library from eight soy proteins. Of the 42 DCA‐binding peptides, 41 exhibited significant degradation of DCA micelles. Peptides capable of specifically disrupting DCA micelles were present in soy pepsin hydrolysates.

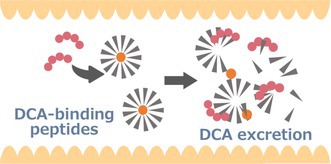

## 1 Introduction

Orally administered dietary components are digested in the stomach and intestines, and primarily absorbed in the intestine. Lipophilic components, such as fatty acids and cholesterol, are insoluble and not easily absorbed. However, bile acids secreted into the gastrointestinal tract form micelles that solubilize them, allowing their absorption from the intestine (Stellaard and Lütjohann 2021).

Bile acids are synthesized from cholesterol in the liver, and primary bile acids, such as cholic acid (CA) and chenodeoxycholic acid (CDCA), are biosynthesized in the bodies of many mammals. The generated primary bile acids are secreted as taurine‐ or glycine‐conjugated bile acids, solubilized, and released into the duodenum via gallbladder contractions. Bile acid micelles containing lipophilic components are absorbed in the upper small intestine and colon, and return to the liver through the portal vein. This process is called enterohepatic circulation, and the bile acids circulating therein account for up to 95% of the bile acids secreted into bile. Bile acids are deconjugated and dehydroxylated by intestinal bacteria, converting them into secondary bile acids, such as deoxycholic acid (DCA) and lithocholic acid. Among secondary bile acids, DCA is known to be a risk factor for colorectal and liver cancers; therefore, its excretion from the body is desirable, leading to the development of functional food materials with important effects (Gill and Rowland 2002).

Soybean ( *Glycine max* ) is the most economically important legume globally and provides plant‐based proteins to millions of people. Soy protein is a relatively inexpensive protein source that contains all essential amino acids found in animal proteins, making its nutritional value nearly equivalent to that of high‐quality animal‐derived proteins (Singh et al. 2014). Additionally, it has demonstrated functional properties including antihypertensive, anti‐cholesterol, antioxidant, and anticancer effects. Bioactive peptides were found in enzymatic hydrolysates of soy proteins. Kim et al. isolated a hydrophobic nonapeptide exhibiting anticancer activity from soybean protein hydrolysates, prepared using thermoase (Kim et al. 2000). Yi et al. (2020) identified a peptide derived from enzymatically hydrolyzed SBP that inhibits apoptosis in the macrophage‐like cell line, RAW264.7, by inducing the expression of anti‐apoptotic proteins (Yi et al. 2020). Wang et al. (2019) reported the identification of α‐glucosidase inhibitory peptides from SBP hydrolysates treated with alkaline protease.

Soy proteins take longer to digest and are absorbed more slowly than animal proteins, such as whey or egg proteins. Since Carroll and Hamilton first reported the effects of soy proteins on lowering plasma LDL levels in 1975 (Carroll and Hamilton 1975), many studies have been conducted, particularly from the perspective of inhibiting cholesterol absorption and improving lipid metabolism. Sugano et al. proposed a mechanism in which highly undigested high molecular weight fraction (HMF) bind to excessive bile acids in the intestine and are excreted from the body (Sugano et al. 1990; Gatchalian‐Yee et al. 1997). Based on the active physiological regulatory functions of soy protein, the US Food and Drug Administration has recognized soy protein as a “food that reduces the risk of coronary heart disease,” due to its cholesterol‐lowering effect (Food and Drug Administration 1999). Regarding the triglyceride‐regulating effect of soy protein, there are reports of significantly reduced serum and liver triglyceride concentrations compared to that with casein (Aoyama, Fukui, Nakamori, et al. 2000). Long‐term soy protein intake is also expected to reduce body weight and fat content, and reduce obesity (Aoyama, Fukui, Takamatsu, et al. 2000). Research has also been conducted on bile acid‐binding peptides derived from soy protein. Peptides showing strong binding activity to taurocholic acid (TCA), which plays the most important role in fatty acid and cholesterol absorption, have been identified, such as the six‐residue peptide VAWWMY derived from β‐conglycinin (Choi et al. 2004). The peptide has been reported to have cholesterol‐lowering effects (Nagaoka et al. 2010). Bile acid‐binding peptides precipitate cholesterol by breaking down bile acid micelles and inhibiting their absorption, leading to decreased serum cholesterol levels. Thus, soy proteins significantly contribute to human health.

Previously, we synthesized peptide libraries on peptide arrays to obtain bioactive peptides, explored high‐binding peptides for TCA, and discovered peptides with even higher binding affinity for TCA than for VAWWMY, using machine learning (Takeshita et al. 2011). Combined with an edible protein database, peptides that bind to TCA are present in various edible proteins (Imai, Shimizu, et al. 2021). However, we did not explore secondary bile acid‐binding peptides, and to the best of our knowledge, research on secondary bile acid‐selective excretion, including that of other groups, has not yet been performed.

In this study, we focused on secondary bile acids, which are risk factors for cancer and are excreted from the body, and attempted to identify peptides derived from soy proteins that strongly bind to DCA and exhibit DCA micelle‐disrupting activity. First, we synthesized a six‐residue peptide library consisting of 1,391 peptides based on the protein sequence and explored high‐binding peptides. Next, we synthesized the free peptides and evaluated their micelle‐disrupting activity. We constructed a 96‐well high‐throughput assay system that simultaneously evaluated micelle‐disrupting activity using four types of bile acids and searched for peptides that specifically disrupted DCA micelles. Subsequently, we analyzed soy pepsin hydrolysates (SPHs) using liquid chromatography–tandem mass spectrometry (LC–MS/MS), identified peptides in the hydrolysate, and evaluated their micelle‐disrupting activity. Furthermore, an undigested HMF was prepared, solubilized with urea, and enzymatically processed to create solubilized samples. Mass spectrometry revealed the presence of three DCA‐binding peptides in HMF. These findings suggested that the peptides identified in this study have the potential to promote the excretion of secondary bile.

## 2 Materials and Methods

### 2.1 Materials

The Fmoc amino acids were purchased from Watanabe Chemical Industries Co. Ltd. (Hiroshima, Japan). Bovine serum albumin (BSA, 019–15123), cholesterol (038–03005), oleic acid (159–00246), cholesterol kit (439–17501), casein powder (030‐01505) and sodium cholate (CA, 197–08502) were purchased from Fujifilm Wako Pure Chemical Corporation (Osaka, Japan). Sodium taurocholate (TCA, T4009) and sodium taurodeoxycholate (TDCA, T0875) were purchased from Sigma‐Aldrich (St. Louis, MO, USA). Sodium deoxycholate (DCA, C0316) was purchased from Tokyo Chemical Industry Co. Ltd. (Tokyo, Japan). Monoolein (23408–12) and egg‐derived phosphatidylcholine (27554–01) were purchased from Nacalai Tesque Inc. (Kyoto, Japan). The anti‐DCA antibody (PAS089Ge01) was purchased from Cosmo Bio Co. Ltd. (Tokyo, Japan). Alexa Fluor 488‐conjugated anti‐rabbit IgG antibody (ab150077) was purchased from Abcam (Cambridge, UK).

### 2.2 Peptide Array Synthesis

To comprehensively investigate bioactive peptides from soy proteins, peptide libraries of six residues were designed with a three‐residue shift from eight SBPs: beta‐conglycinin alpha subunit 1 (7S_alpha, P0DO16), beta‐conglycinin alpha’ subunit (7S_alphaP, P11827), beta‐conglycinin beta subunit 1 (7S_beta, P25974), glycinin G1 (11S_A1aB1b, P04776), glycinin G3 (11S_A1aB2, P11828), glycinin G2 (11S_A2B1a, P04405), glycinin G5 (11S_A3B4, P04347), and glycinin G4 (11S_A5A4B3, P02858). A total of 1,391 peptide sequences were designed, as listed in Table S1, and synthesized via solid‐phase synthesis following a previous report (Kozaki et al. 2017). Each peptide was synthesized on a cellulose membrane activated with β‐alanine using a spot synthesizer (ASP222, Intavis, Köln, Germany), and Fmoc‐Aund(11)‐OH (K00962, Watanabe Chemical Industries Co. Ltd.) was introduced as a spacer at the C‐terminus of the peptide. An Fmoc amino acids of 0.3 mol/L was used for peptide synthesis. At each elongation step, each amino acid was spotted three times to prevent retention of unreacted molecules. After synthesis, the side‐chain protecting groups of Fmoc amino acids were deprotected. The membrane was thoroughly washed with diethyl ether and methanol, and incubated with phosphate‐buffered saline (PBS). All peptides were synthesized in triplicates.

### 2.3 Bile Acid Binding Assay

A bile acid‐binding assay was conducted with modifications using antibodies against DCA as described previously (Imai, Shimizu, et al. 2021). After washing the prepared peptide array with PBS, it was immersed in 10 μg/mL DCA solution dissolved in PBS and incubated at 37°C for 1 h under mild agitation. After washing with PBS, the array was incubated at 37°C for 1 h under mild agitation in an anti‐DCA antibody solution in PBS containing 0.25% BSA at a concentration of 2.5 μg/mL. After washing with Tris‐buffered saline containing 0.05% Tween 20 (T‐TBS, pH 7.4), the array was incubated with a solution of Alexa 488‐labeled anti‐rabbit IgG antibody dissolved in PBS at a concentration of 4 μg/mL at 37°C for 1 h under mild agitation. The peptide array was washed with T‐TBS and captured using a Typhoon FLA 9500 biomolecular imager (GE Healthcare Life Sciences, Japan). The emission and excitation wavelengths were 495 and 519 nm, respectively. ImageQuant TL software (GE Healthcare Life Sciences) was used for analysis and the fluorescence intensity of each peptide spot was quantified. To eliminate nonspecific secondary antibody adsorption, a separate peptide array was prepared and incubated with only secondary antibodies, without primary antibodies, and fluorescence intensity was quantified. Subsequently, the fluorescence intensity without the primary antibodies was subtracted from that with the primary antibodies. The same peptide was spot‐synthesized in triplicate, therefore, the average of subtracted fluorescence intensity of three spots was calculated. This value, “fluorescence intensity [a.u.],” was defined as the binding to DCA.

### 2.4 Peptide Resin Synthesis

Peptide resin synthesis was performed using the Fmoc solid‐phase peptide synthesis method, according to a previous report (Imai, Takeuchi, et al. 2021). The synthesis was performed using a polypropylene column (2600030, Kokusan Chemical Co. Ltd., Tokyo, Japan) and Fmoc‐amino acid‐alko resin (Watanabe Chemical Industries Co. Ltd.). First, the Fmoc groups were removed by adding 20% piperidine (A00176, Watanabe Chemical Industries Co. Ltd.) to 7 mL of N, N‐dimethylformamide (DMF; 10344–80, Kanto Chemical Co. Inc., Tokyo, Japan), washed three times with DMF, and the resin was subjected to an elongation reaction by mixing three equivalent amounts of 2‐(1H‐benzotriazole‐1‐yl)‐1,1,3,3‐tetramethyluronium hexafluorophosphate (A00149, Watanabe Chemical Industries Co. Ltd.) and N, N‐diisopropylethylamine (D1599, Tokyo Chemical Industry Co. Ltd.) with Fmoc amino acid‐OH in DMF (5 mL per amino acid mole equivalent). After washing with DMF and methanol, the elongation was repeated. After synthesis, the Fmoc groups were removed from the amino acid side chains and the peptides were cleaved from the resin. The peptide was precipitated by adding diethyl ether (14134–81, Kanto Chemical Co. Inc.) and freeze‐dried to obtain a dry powder.

### 2.5 Protein Hydrolysis

Pepsin hydrolysis of casein powder was performed as previously described (Hagawa et al. 2022). Three grams of casein powder was dissolved in 60 mL of ultrapure water and homogenized. The pH of the solution was adjusted to 2.0 by adding 1 M HCl (080–01066, Fujifilm Wako Pure Chemical Corporation). Then, 375 mg of pepsin (161–24482, Fujifilm Wako Pure Chemical Corporation) was added and incubated at 37°C for 5 h. This hydrolysis time corresponds to the duration of enzymatic hydrolysis reported by Sugano et al. (1988) for obtaining undigested HMF of SBP (Sugano et al. 1988). The pH was adjusted to 7.0 by adding 1 M NaOH (195–13775, Fujifilm Wako Pure Chemical Corporation) to stop the reaction. The supernatant was collected by centrifugation at 12,000 × *g* at 4°C for 30 min and freeze‐dried.

### 2.6 Bile Acid Micelle Disruption Assay

To improve the throughput, a 96‐well assay system was developed for bile acid micelle disruption based on a previous study (Ito et al. 2020). Bile acid micelles were prepared as described by Nagaoka et al. (1999). Cholesterol (3.89 mg), oleic acid (9.7 μL), monoolein (37.9 μL), and phosphatidylcholine (8.81 mg) were mixed as the lipid components and dissolved in methanol. After evaporation of the solvent, the resulting lipid film was mixed well with 10 mL of a single bile acid (DCA, CA, TDCA, or TCA) at a concentration of 13.18 mmol/L by sonication and incubated at 37°C for 2 h. The PBS (pH 7.4) containing bile acids (DCA, CA, TDCA, and TCA, all at 3.3 μmol/L each in PBS, pH 7.4) was used to dissolve the peptide, and the peptide concentration was adjusted to 12 mg/mL. The bile acid micelle and peptide solutions were added to a V‐bottom 96‐well plate (BM6002, BMS Corporation, Tokyo, Japan) at a ratio of 1:1, 100 μL each, and mixed. The final concentrations of each component in the bile acid micelle solution were 6.6 mmol/L of target bile acids, 1.65 μmol/L of the other three bile acids, 0.5 mmol/L of cholesterol, 1.5 mmol/L of oleic acid, 5 mmol/L of monoolein, 0.6 mmol/L of phosphatidylcholine, and 6 mg/L of peptide. Bile acids (10 mM) are usually present in the small intestine of healthy humans (Ticho et al. 2020); therefore, 6.6 mmol/L of bile acid was used in the micelle disruption assay as described in previous reports (Nagaoka et al. 1999; Ito et al. 2020; Imai, Takeuchi, et al. 2021; Iriyama et al. 2024). Bile acid micelle disruption activity was evaluated by measuring the micelle solubility of cholesterol. In the absence of micellar disruption, cholesterol remains 100% soluble. Lower solubility indicates greater micelle disruption. After incubation at 37°C for 2 h under rotary stirring, the plate was centrifuged at 2,850 × g at 37°C for 20 min. The micelle cholesterol exists in the supernatant, not the precipitate. Therefore, the supernatant was collected, filtered through a 0.45‐μm filter, and then used to measure cholesterol concentration in the micelles. To measure cholesterol concentration in the micelles existing in the supernatant, 40 μL of the supernatant was mixed with 240 μL of cholesterol kit solution in a 96‐well plate (3860–096, AGC Techno Glass Co. Ltd., Shizuoka, Japan). After incubation at 37°C for 3 min, the absorbance was measured at 600 nm using an EPOCH 2 absorbance plate reader (Waken Btech Co. Ltd., Kyoto, Japan). The percentage of cholesterol in the micelles was defined as the micellar solubility of cholesterol, which was calculated by subtracting the absorbance of the sample from the absorbance of the control without peptide, divided by the absorbance of the control without peptide. All experiments were performed in triplicate.

### 2.7 Multiple Regression Analysis

Multiple regression analysis was performed using the cholesterol solubility of the peptides tested in the bile acid micelle disruption assay as objective variables. The average values of seven indicators of each residue, namely isoelectric point, polarity, hydrophobicity, molecular weight, α‐helical propensity, β‐turn propensity, and count of aromatic amino acids, were initially used as explanatory variables. Next, 24 indicators for each residue position were added instead of the average values of the four amino acid indicators: isoelectric point, polarity, hydrophobicity, and molecular weight. Considering the effect of multicollinearity, the polarity from the second to sixth residue (residue 2 to 6_polarity) was removed from the explanatory variables if the Correlation Coefficient between the explanatory variables was |correlation coefficient| ≥ 0.8 and the average polarity was added. The explanatory variables comprised 23 indicators. Multiple regression analyses were performed for each bile acid using these variables. In this analysis, non‐significant variables with *p*‐values of 0.05 or higher were incorporated into the error variance, and coefficient estimates were scaled so that each variable had a mean of zero and a range of two.

### 2.8 LC–MS/MS Analysis

According to a previous study (Iriyama et al. 2024), LC–MS/MS analysis of the resultant peptides was performed using a UHPLC connected to a Q‐Exactive Orbitrap mass spectrometer (Thermo Fisher Scientific, Waltham, MA, USA) through a nanoelectrospray ion source (AMR Inc., Tokyo, Japan). The desalted peptides were loaded onto a separation capillary C18 reverse‐phase column (NTCC‐360/100–3‐125, 125 × 0.1 mm, Nikkyo Technos, Japan). The eluents used were A (100% water containing 0.5% acetic acid) and B (80% acetonitrile containing 0.5% acetic acid), and the column was operated at a flow rate of 0.5 μL/min with a gradient of acetonitrile concentration. The mass spectrometer was operated in data‐dependent acquisition mode. The analysis conditions were set as follows: capillary temperature: 250°C; capillary voltage: 2.0 kV in positive mode, S‐lens RF level: 50. The MS1 spectra were measured at a resolution of 70,000, an automatic gain control (AGC) target of 3 × 10^6^, and a mass range of 350–1,800 m/z. HCD MS/MS spectra were acquired with a resolution of 17,500, an AGC target of 1 × 10^5^, an isolation window of 2.0 m/z, a maximum injection time of 60 ms, and a normalized collision energy of 27. Dynamic exclusion was set to 20 s. The MS spectra of the peptides were recorded using an Xcalibur 3.0.63 system (Thermo Fisher Scientific). MS/MS spectra were used to create a peak list using Proteome Discoverer 2.2.0.388 (Thermo Fisher Scientific) and peptide sequences were searched using SEQUEST (Thermo Fisher Scientific) based on a significant Xcorr value (high‐confidence filter).

### 2.9 Statistical Analysis

All experiments were performed in triplicates. Student's *t*‐test was used to evaluate statistical significance.

## 3 Results

### 3.1 Fabrication of a 6‐Residue Soy Protein Library and Screening of DCA Binding Peptides

**FIGURE 1:**
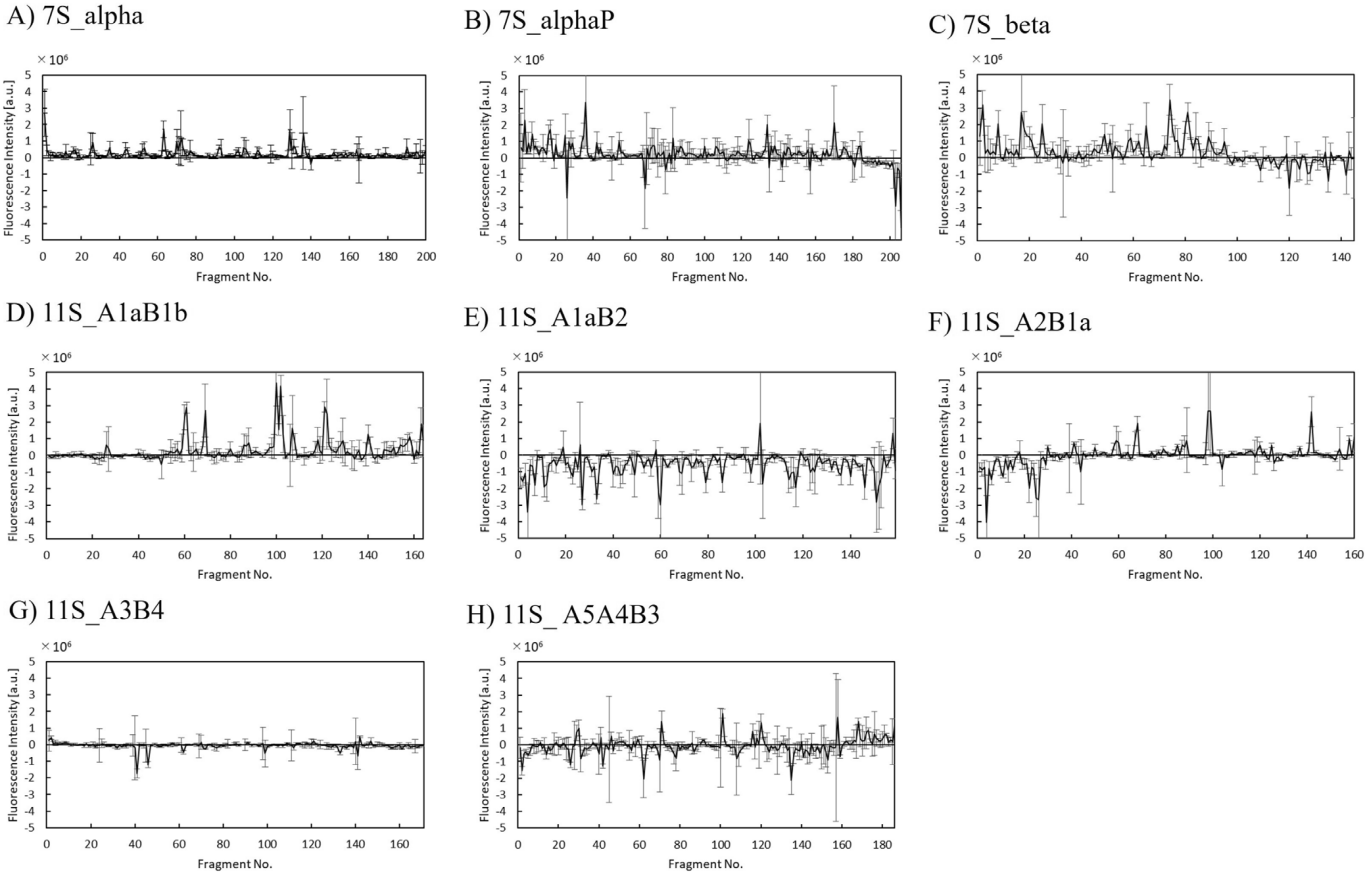
DCA binding activity of 1,391 peptides from 8 soy protein subunits. All values are shown as the mean ± SD (*n* = 3). (A) 7S_alpha: Beta‐conglycinin alpha subunit 1 (P0DO16), (B) 7S_alphaP: Beta‐conglycinin alpha’ subunit (P11827), (C) 7S_beta: Beta‐conglycinin beta subunit 1 (P25974), (D) 11S_A1aB1b: Glycinin G1 (P04776), (E) 11S_A1aB2: Glycinin G3 (P11828), (F) 11S_A2B1a: Glycinin G2 (P04405), (G) 11S_A3B4: Glycinin G5 (P04347) and (H) 11S_A5A4B3: Glycinin G4 (P02858).

To comprehensively investigate DCA‐binding sites within soy proteins, a peptide array containing 1,391 sequences was synthesized as a peptide array for eight soy protein subunit sequences and sectioned into hexamer peptide fragments with a three‐residue shift from the N‐terminus. A bile acid‐binding assay was conducted using DCA, a DCA antibody, and fluorescently labeled secondary antibodies. The fluorescence intensity of each protein subunit is shown in Figure 1, and the sequences are listed in Table S1. A comparison of the fluorescence intensities of the protein subunits is shown in Table 1. The average fluorescence intensities were high for 7S_beta and 11S_A1aB1b. Due to the high variability in binding evaluation experiments using antibodies, we selected high‐binding sequences based on the average and standard deviation *σ* of fluorescence intensity. Those values for all sequences were average = 4.25 × 10^4^ [−] and *σ* = 6.56 × 10^5^ [−]. Sequences with fluorescence intensity ≥ 2.0 of the *Z*‐score (average + 2*σ* ≥ 1.35 × 10^6^ [−]) were defined as DCA high‐affinity sequences. Previously, we used this criterion to define and select “high‐binding sequences” in peptide arrays and found some bioactive peptides based on this criterion and published in academic papers (Imai, Shimizu, et al. 2021). Among the 1,391 sequences, 42 met this criterion. A list of the 42 peptides is provided in Table S2. There were 11 sequences of 7S_beta and 10 sequences of 11S_A1aB1b, accounting for half of all the sequences. Moreover, when comparing sequences with slightly high intensity and a *Z*‐score from 1.0 to 2.0, there were 20 sequences for 7S_alphaP and 18 sequences for 7S_beta, comprising more than half of the total. This suggests that 7S_beta and 7S_alphaP are subunits with high affinity for DCA.

**TABLE 1:** Comparison of protein subunits on DCA binding.

Protein	Peptides	Average intensity	No. of peptides with *Z*‐score^a^ ≧ 2.0	No. of peptides with 2.0 > *Z*‐score^a^ ≧ 1.0
7S_alpha	200	203,314	4	3
7S_alphaP	206	206,968	8	20
7S_beta	145	323,903	11	18
11S_A1aB1b	164	300,089	10	9
11S_A1bB2	159	−588,941	1	1
11S_A2B1a	160	−40,891	4	7
11S_A3B4	171	−53,927	0	0
11S_A5A4B3	186	−58,925	4	7
Total	1,391	42,497	42	65

^a^ *Z*‐score = 2.0 and 1.0 mean total average + 2*σ* and total average + *σ*, respectively.

**FIGURE 2:**
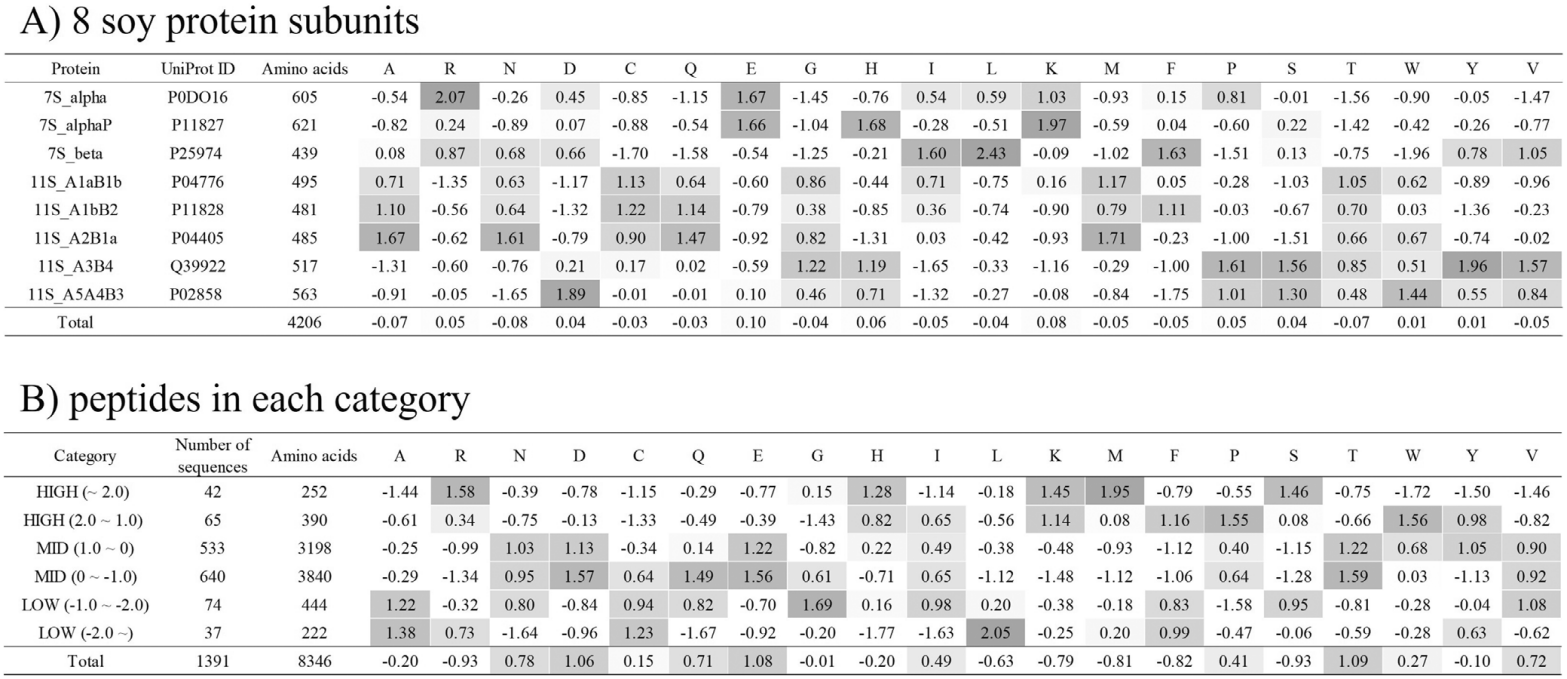
*Z*‐score of amino acid contents of 8 soy protein subunits (A) and peptides in each category (B). *Z*‐score was calculated from the mean and standard deviation of the content of each amino acid residue using the following formula: (A) *Z*‐score of Ala content = (Ala content of protein of interest—Average of Ala content of 8 soy protein subunits)/(SD of Ala content of 8 soy protein subunits), (B) *Z*‐score of Ala content = (Ala content of category of interest—Average of Ala content of 6 categories)/ (SD of Ala content of 6 categories). Both are represented in a heatmap according to the deviation score of abundance, with gray indicating higher proportions compared with other proteins (Figure 2A) or other peptide groups (Figure 2B).

Furthermore, the proportions of amino acid residues for each protein subunit and the proportions of amino acid residues categorized based on the DCA‐binding affinity of the peptides were investigated (Tables S3A and S3B). Based on these data, the *Z*‐scores of the amino acid residue proportions for the eight protein subunits or six categories based on the DCA‐binding affinity of the peptides were calculated, as described in Figure 2. Both 7S_alpha and 7S_alphaP had high proportions of acidic (E) and basic (R) amino acids. The protein subunit 7S_beta had a high content of hydrophobic amino acid residues I, L, and V, and a high proportion of R residues (Figure 2A). In categories with high binding strength, such as high *Z*‐scores, R and K were abundant, whereas E and D were less abundant (Figure 2B). This was consistent with both the abundance of R residues and the lack of E residues in 7S_beta, which showed a high DCA binding affinity. This suggests that the positively charged residues contribute to DCA binding, highlighting the importance of basic amino acids.

### 3.2 Validation Using a Bile Acid Micelle Disruption Assay

**FIGURE 3:**
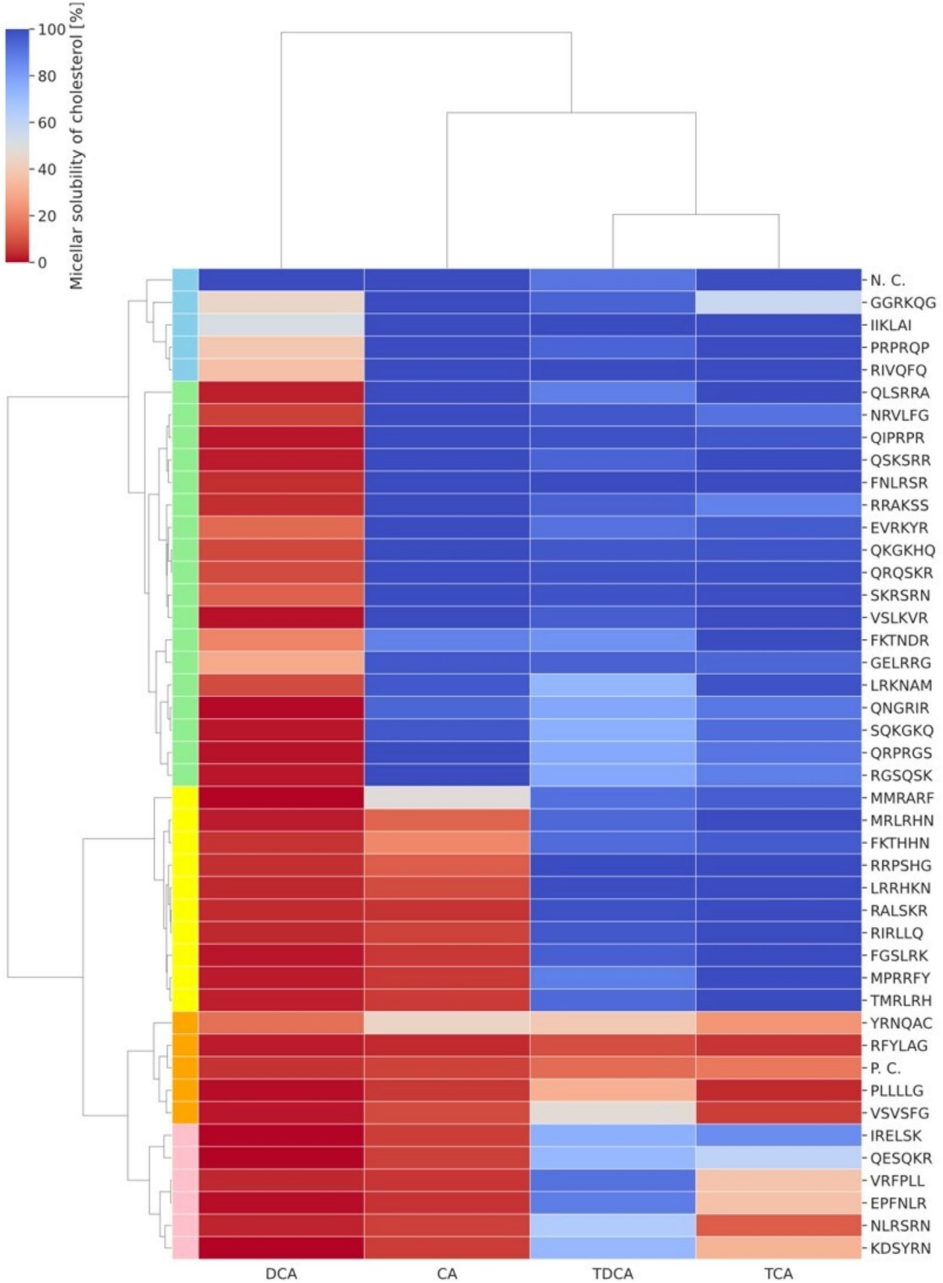
Clustering results based on DCA micelle disrupting activity of 42 peptides. All experiments were performed in triplicate. CA, cholic acid; DCA, deoxycholic acid; TCA, taurocholic acid; TDCA, taurodeoxycholic acid.

To verify the activity of the 42 sequences selected for the bile acid binding assay, their ability to act as free peptides against DCA was examined using the bile acid micelle disruption assay. By expanding the types of bile acids from DCA to include CA, TDCA, and TCA, differences in the effectiveness of DCA high‐binding peptides were compared among bile acids. The 42 sequences were synthesized on a resin, and the synthesized peptides were subjected to a bile acid micelle disruption assay, along with a positive (cholestyramine) and negative control (casein pepsin hydrolysate) (Figure 3, Table S4). Clustering revealed that the data can be divided into five groups. These groups included 4 sequences (sky blue) showing slight activity towards DCA micelles; 18 sequences (light green) showing activity only towards DCA; 10 sequences (yellow) showing activity towards both DCA and CA; 4 sequences (orange) showing activity towards all four bile acids; and 6 sequences (pink) showing activity towards DCA and CA, and slight activity towards TCA. Focusing on the DCA micelle column, it was verified that except for one sequence (IIKLAI), all 41 DCA high‐binding peptides exhibited micelle disruption activity against DCA, with a cholesterol solubility rate ≤ 50%. Furthermore, the 10 peptides with higher intensities in the DCA‐binding assay showed relatively high activity.

### 3.3 Characterization of DCA Micelle Disruption Peptides

**FIGURE 4:**
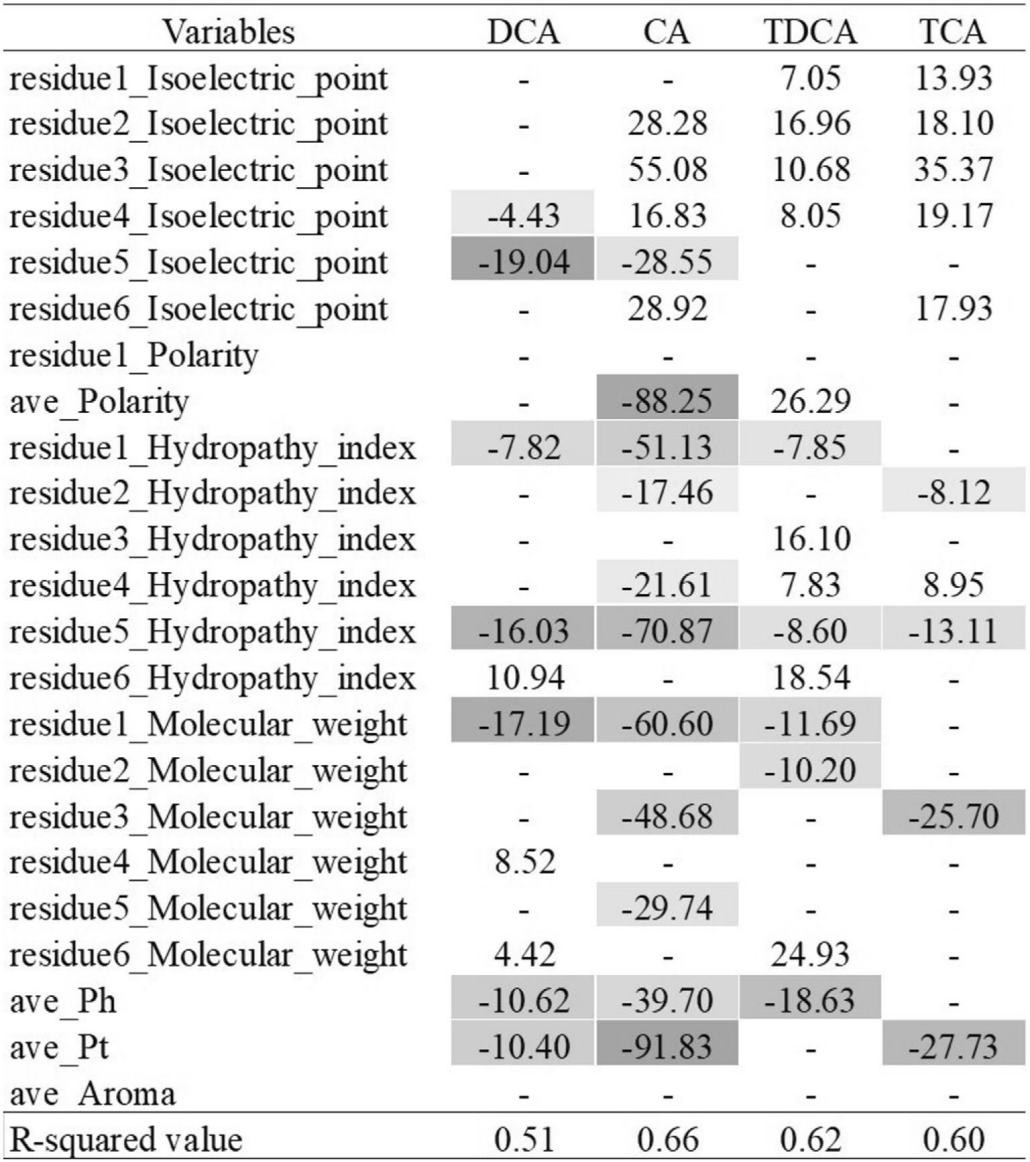
Scaled estimated values in multiple linear regression analysis using 23 explanatory variables for each 4 bile acids. Aroma, count of aromatic amino acids; Ave, Average; Ph, α‐helical propensity; Pt, β‐turn propensity.

Based on the results of bile acid micelle disruption tests, a sequence function analysis was conducted. Data from *n* = 3 for 42 sequences against the four bile acids were used as objective variables. Initially, multiple linear regression analysis was performed for each of the four bile acids using the six‐residue averages of seven amino acid indices: isoelectric point, polarity, hydrophobicity, molecular weight, α‐helical propensity, β‐turn propensity, and number of aromatic amino acids. However, the determination coefficients were very small, suggesting a low predictive accuracy (Figure S1). Therefore, in the next step, we used the values at each residue position for the four important amino acid indices (isoelectric point, polarity, hydrophobicity, and molecular weight), instead of the average values, resulting in 24 indices as explanatory variables. To avoid destabilizing the model owing to multicollinearity, the correlation between the 24 indices was examined (Figure S2). Polarity was found to correlate with the isoelectric point and hydrophobicity; therefore, only the polarity of the first residue was retained and the others were removed. Using the value at each position yielded average dependent variables; therefore, the average isoelectric point, hydrophobicity, and molecular weight were removed, and multiple linear regression analysis was conducted using 23 explanatory variables. Models with R‐squared values > 0.5 were obtained. Figure 4 presents the scaled estimated values of each explanatory variable. For DCA, the scaled estimated values of the isoelectric points at the fourth and fifth residues were negative. This indicates that a high isoelectric point at the fourth or fifth residue (e.g., K and R) correlates with high DCA micelle disruption activity. In contrast, for the other bile acids, a high isoelectric point at any residue, except for the fifth, did not contribute to micelle disruption. The scaled estimated values were positive, indicating that a low isoelectric point (e.g., D and E) correlated with micelle disruption. Conversely, a common feature of all four bile acids is that the high hydrophobicity at the fifth residue makes micelle disruption more likely. Additionally, the scaled estimated values of Ph (α‐helical propensity) and Pt (β‐turn propensity) were negative for all four bile acids, indicating that sequences with helicity and turning tendency in the amino acid residues of the peptide are likely to disrupt micelles. Focusing on the tauroconjugates, TCA and TDCA, the scaled estimated hydrophobicity of the fourth residue was positive. In contrast, the same index has a negative value for CA. The tauroconjugate group is heavily influenced by electrostatic interactions owing to the charge of the taurine moiety; therefore, it is less susceptible to hydrophobicity‐induced micelle disruption. Conversely, bile acids that are not conjugated are likely to be affected by hydrophobic‐peptide‐induced micelle disruption on the hydrophobic surface of the sterol skeleton.

### 3.4 Identification of Soy Hydrolysate Peptide Sequences and Evaluation of DCA Micelle Disruption Activity Using Synthetic Peptides

**FIGURE 5:**
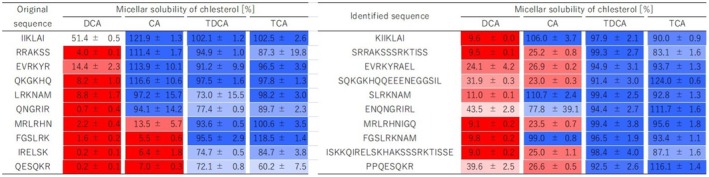
Bile acid micelle disruption activity of identified peptides existing in SPH. All values are shown as the mean ± SD (*n* = 3). Original sequence means the sequence of 6‐mer peptides selected by a DCA binding assay. Identified sequence means the sequence of peptides containing the “original sequence”, as identified by LC–MS/MS.

To investigate whether SPHs contained fragments that included high‐activity DCA micelle disruption sequences, peptide identification was performed using LC–MS/MS. A total of 1,354 sequences were identified. Among them, 10 sequences were discovered from a pool of 42 sequences upon examination of a small fragment containing DCA high‐binding six‐residue sequences. Bile acid micelle disruption assays were conducted using the peptides synthesized on the resin (Figure 5). Focusing on DCA micelles, compared to the original sequences that are the sequences of the peptide array, all peptides exhibited cholesterol solubility < 50% in DCA micelles, indicating that their ability to disrupt DCA micelles was retained.

SBPs produce undigested fractions during enzymatic digestion, which resist enzymatic activity and are subsequently excreted in the feces. To investigate these fractions, we prepared the undigested fractions, solubilized them with urea, and subjected them to enzymatic digestion with Lys‐C and Glu‐C. The peptides in the resulting samples were identified using LC–MS/MS analysis. This analysis detected five peptides corresponding to four of the 42 high‐DCA‐binding sequences (Table S5). Among these, FGSLRKNAM and SLRKNAM were included (Figure 5).

In this study, we successfully identified 10 novel peptides with DCA micelle disruption activity in SPHs. These 10 sequences are believed to be peptides that promote the in vitro excretion of secondary bile acids. Notably, FGSLRKNAM and SLRKNAM selectively disrupted DCA micelles without affecting other bile acid micelles and were detected in the indigestible fractions. This suggests that the administered peptides bind specifically to DCA, facilitating the disruption of DCA micelles and promoting their excretion, thereby offering an efficient mechanism for DCA elimination.

## 4 Discussion

The DCA‐binding sequence was evaluated using peptides synthesized in the solid phase from the eight soy protein subunits. When comparing the DCA high‐binding sequences for each protein, we observed that 7S_alpha, 7S_beta, and 11S_A1aB1b had a higher number of DCA high‐binding sequences, whereas 11S_A1bB2 and 11S_A3B4 showed fewer prominent peaks. Proteins with a high number of DCA high‐binding sequences predominantly contained hydrophobic amino acids, such as Ile and Leu, as well as basic amino acids, such as Lys and Arg. Our results are in accordance with those of our previous research on peptides interacting with TCA, a primary bile acid, in which peptides containing basic residues were identified as high‐binding peptides (Imai, Shimizu, et al. 2021).

The bile acid micelle disruption assay of the 42 sequences resulted in their classification into five clusters. The four bile acids evaluated share a common structural feature, in which hydroxyl and carboxyl groups exist on the alpha face of the sterol skeleton and the beta face becomes hydrophobic (Mukhopadhyay and Maitra 2004). Differences were observed based on the number of hydroxyl groups and the presence of taurine conjugates. Therefore, based on these structural variations, it is suggested that DCA‐selective binding peptides act through multiple binding sites based on these structural variations, not through a simple binding site. The negatively charged carbonyl and sulfonic acid groups in the four bile acids interact electrostatically with basic amino acid residues, contributing to the disruption of bile micelles. However, the high isoelectric points of TCA, TDCA, and CA contributed to the micelle stabilization. Taurine‐conjugated TCA and TDCA, owing to their lower pKa values, do not disrupt micelles solely through positive charges but may require hydrophobic interactions from highly hydrophobic amino acid residues or electrostatic repulsion from an increased number of negatively charged residues. In particular, for CA, the higher hydrophobicity at all residue positions indicated a higher propensity for micelle disruption. In addition, CA has one more hydroxyl group than DCA. Hydrogen bonding interactions are essential for disrupting bile acid micelles, which are formed through hydrogen bonds in peptide secondary structures, such as helices and turns. The scaled estimates of Ph and Pt were negative for all bile acids, suggesting that sequences prone to secondary structure formation are more likely to disrupt micelles, particularly in CA.

A comparison of the long‐chain peptides identified from SPH with the corresponding six‐residue peptides revealed three new long‐chain peptides exhibiting CA micelle disruption activity, whereas one lost CA micelle disruption activity. Among the three newly active peptides, one had abundant Ser residues and two had Leu residues at the C‐terminus, accompanied by an increase in Glu residues. It was suggested that this increased the hydrogen bonding. The peptide losing CA micelle disruption activity of FGSLRKNAM showed DCA micelle‐specific disruption. The peptide contained an additional NAM sequence at the C‐terminus, coinciding with the absence of CA micelle disruption activity at the C‐terminus of LRKNAM. The addition of NAM likely eliminates the positive charge at the C‐terminus, resulting in the loss of CA micelle disruption activity.

## 5 Conclusion

In this study, we synthesized a six‐residue peptide library from eight soy protein sequences and evaluated the DCA‐binding activities of 42 candidate peptides. A high‐throughput 96‐well assay system was developed to assess the disruption activity of bile salt micelles against four types of bile salts. Evaluation of the 42 free peptides revealed strong DCA micelle‐disruption activity in 41 sequences. Analysis of sequence characteristics, considering other bile salt micelle disruption activities, suggested contributions from the positive charge, hydrophobicity, and ease of secondary structure formation. Furthermore, pepsin‐hydrolyzed soy protein was prepared, and LC–MS/MS analysis identified 1,354 free peptides. Comparison of these identified peptides with the 42 peptides tested for DCA‐binding activity led to the selection of 10 peptides that were chemically synthesized and evaluated for DCA micelle‐disrupting activity. All 10 peptides exhibited activity.

These peptides are believed to promote secondary bile excretion. FGSLRKNAM and SLRKNAM selectively disrupted DCA micelles, suggesting their potential for efficient DCA excretion. Additionally, these two peptides were identified in the indigestible fraction, highlighting their importance in facilitating DCA excretion.

## Author Contributions

**Shota Shimizu:** formal analysis (lead), investigation (lead), methodology (lead), validation (equal), writing – review and editing (equal). **Keita Hirano:** investigation (supporting), writing – review and editing (supporting). **Tsutomu Saito:** supervision (equal), writing – review and editing (supporting). **Hirokazu Akiyama:** supervision (equal), writing – review and editing (equal). **Kazunori Shimizu:** supervision (equal), writing – review and editing (supporting). **Hiroyuki Honda:** funding acquisition (lead), project administration (lead), supervision (lead), writing – original draft (lead), writing – review and editing (equal).

## Conflicts of Interest

The authors declare the following financial interests/personal relationships that may be considered as potential competing interests: H.H., K.S., and H.A. received financial support from the JSPS, and H.H. also did from the Research Institute for Creating the Future in Fuji Oil Holdings Inc. Authors K.H. and T.S. are employees of Fuji Oil Holdings Inc. There are no other conflicts of interest to declare.

## Supporting information


Data S1.

## Data Availability

Data available on request from the authors.
