# Supplementary material for: Identification of Soy‐Derived Peptides With Micelle Disruption Activity of Secondary Bile Acids
Source: Food Sci Nutr. 2025 May 26;13(6):e70319. doi: 10.1002/fsn3.70319 (PMC12121512; doi:10.1002/fsn3.70319)
Supplement: Supplementary file 1 — Data S1. [file FSN3-13-e70319-s001.docx]

**Supplementary information**

Title of manuscript:

Identification of soy-derived peptides with micelle disruption activity of secondary bile acids

Authors:

Shota Shimizu 1, shimizu.shota.1106@gmail.com

Keita Hirano 2, hirano.keita@so.fujioil.co.jp

Tsutomu Saito 2, saito.tsutomu@so.fujioil.co.jp

Hirokazu Akiyama 1, hirokazu.akiyama@chembio.nagoya-u.ac.jp

Kazunori Shimizu 1, shimizu@chembio.nagoya-u.ac.jp

Hiroyuki Honda 1, honda@chembio.nagoya-u.ac.jp

Affiliation:

1 Department of Biomolecular Engineering, Graduate School of Engineering, Nagoya University, Furo-cho, Chikusa-ku, Nagoya 464-8603, Japan

2 Research Institute for Creating the Future, Fuji Oil Holdings Inc., 4-3, Kinunodai, Tsukubamirai-shi, Ibaraki, 300-2497, Japan

For Table S5, enzymatic hydrolysis for undigested high molecular fraction (HMF) was performed as follows and the resultant solution was analyzed using LC-ESI-QTOF-MS.

The undigested HMF derived from soy protein was prepared according to the method described by Sugano et al. (1988). A 10 wt% solution of soy protein (Fujipro, produced by Fuji Oil Co.) was treated with 0.2 wt% of two enzymes, Bioprase OP (Nagase Viita Co., Okayama, Japan) and Sumizyme FP (ShinNihon Chemical Co., Aichi, Japan), at pH 7 and 50 ℃ for 5 h. After the reaction, the enzymes were inactivated by heating at 100 ℃ for 5 min. The mixture was then cooled to 25 ℃, followed by centrifugation at 8,000 × g for 10 min to obtain the insoluble fraction (HMF), which consisted of undigested proteins.

HMF (1 mg) was solubilized in 1 mL of 50 mM Tris-HCl buffer (pH 8.0) containing 8 M urea. A 200-µL aliquot of the protein solution was treated with the enzyme Lys-C (VA117A: Promega), which specifically cleaves C-terminal to lysine residues, at a protein-to-enzyme ratio of 200:1 (w/w). The reaction was performed at 37 ℃ for 16 h. The enzymatic reaction was terminated by the addition of formic acid (063-05895: FUJIFILM Wako Pure Chemical) to a final concentration of 0.1% (v/v).

Next, dialysis was performed using a Micro Float-A-Lyzer® (MWCO: 0.1–0.5 kDa; F235049: REPLIGEN) to remove urea from the enzymatic reaction mixture against 100 mM phosphate buffer. The dialyzed solution was then treated with Glu-C (V165A; Promega), which specifically cleaves the C-terminus to aspartic acid or glutamic acid residues at a protein-to-enzyme ratio of 200:1 (w/w). The reaction was carried out at 37 ℃ for 16 h and terminated by adding formic acid to a final concentration of 0.1% (v/v).

The resulting protein hydrolysate was analyzed using LC-ESI-QTOF-MS and MS/MS on a Vion IMS QTof system (Waters). The MS range was adjusted to m/z 100–2,000, and the detector voltage was set to 1.0 kV. The obtained MS and MS/MS spectra were processed using the UNIFI Scientific Information System (Waters). Peptide candidates were identified by comparing the data to eight subunits of soy proteins β-conglycinin and glycinin (Uniprot IDs: P0DO16, P11827, P25974, P04776, P11828, P04405, Q39922, and P02858) from UniProt database (https://www.uniprot.org/).
